# Classical cardiovascular disease risk factors associate with vascular function and morphology in rheumatoid arthritis: a six-year prospective study

**DOI:** 10.1186/ar4396

**Published:** 2013-12-02

**Authors:** Aamer Sandoo, Neil Chanchlani, James Hodson, Jacqueline P Smith, Karen M Douglas, George D Kitas

**Affiliations:** 1 Department of Rheumatology, The Dudley Group NHS Trust, Russells Hall Hospital, Pensnett Road, Dudley, West Midlands DY1 2HQ, UK; 2 The School of Sport, Exercise and Rehabilitation Sciences, University of Birmingham, Edgbaston, Birmingham B15 2TT, UK; 3 Wolfson Computer Laboratory, University Hospital Birmingham NHS Foundation Trust, Queen Elizabeth Hospital Birmingham, Mindelsohn Way, Birmingham B15 2WB, UK; 4 Arthritis Research UK Epidemiology Unit, University of Manchester, Oxford Road, Manchester M13 9PT, UK

## Abstract

**Introduction:**

Patients with rheumatoid arthritis (RA) are at an increased risk for cardiovascular disease (CVD). An early manifestation of CVD is endothelial dysfunction which can lead to functional and morphological vascular abnormalities. Classical CVD risk factors and inflammation are both implicated in causing endothelial dysfunction in RA. The objective of the present study was to examine the effect of baseline inflammation, cumulative inflammation, and classical CVD risk factors on the vasculature following a six-year follow-up period.

**Methods:**

A total of 201 RA patients (155 females, median age (25th to 75th percentile): 61 years (53 to 67)) were examined at baseline (2006) for presence of classical CVD risk factors and determination of inflammation using C-reactive protein (CRP) and erythrocyte sedimentation rate (ESR). At follow-up (2012) patients underwent assessments of microvascular and macrovascular endothelium-dependent and endothelium-independent function, along with assessment of carotid atherosclerosis. The CRP and ESR were recorded from the baseline study visit to the follow-up visit for each patient to calculate cumulative inflammatory burden.

**Results:**

Classical CVD risk factors, but not RA disease-related inflammation, predicted microvascular endothelium-dependent and endothelium-independent function, macrovascular endothelium-independent function and carotid atherosclerosis. These findings were similar in a sub-group of patients free from CVD, and not receiving non-steroidal anti-inflammatory drugs, cyclooxygenase 2 inhibitors or biologics. Cumulative inflammation was not associated with microvascular and macrovascular endothelial function, but a weak association was apparent between area under the curve for CRP and carotid atherosclerosis.

**Conclusions:**

Classical CVD risk factors may be better long-term predictors of vascular function and morphology than systemic disease-related inflammation in patients with RA. Further studies are needed to confirm if assessments of vascular function and morphology are predictive of long-term CV outcomes in RA.

## Introduction

Rheumatoid arthritis (RA) is a chronic inflammatory musculoskeletal disease characterised by pain, swelling and stiffness of the joints [1]. Patients with RA also have an increased risk for cardiovascular disease (CVD) compared to the general population [2]. It has been hypothesised that systemic RA-related inflammation exerts deleterious effects on classical CVD risk factors and the vasculature [3], most likely due to similarities between the pathogenesis of RA and atherosclerosis [4].

The endothelium is the innermost layer of the vasculature and is responsible for maintaining an atheroprotective environment within the vessel. Damage to the endothelium from injurious stimuli such as oxidative stress and inflammatory mediators results in endothelial dysfunction, primarily through a reduction in the antiatherogenic molecule nitric oxide (NO) [5]. Several noninvasive assessments of vascular function and morphology can examine different stages of subclinical atherosclerosis and provide useful information on an individual’s CVD risk status.

Laser Doppler imaging (LDI) with iontophoresis of NO agonists is commonly used to assess endothelial function in the microvasculature, whereas flow-mediated dilatation (FMD) (endothelium-dependent) and glyceryl trinitrate–mediated dilatation (GTN) (endothelium-independent) are used to assess macrovascular endothelial function. Assessment of vascular morphology is typically performed using high-resolution B-mode ultrasonography in the carotid arteries, which provides information on the carotid artery intima-media thickness (cIMT) [5]. These assessments are good predictors of future cardiac events in the general population and in patients with CVD [6,7].

Patients with RA have evidence of impaired vascular function and morphology compared to healthy age- and sex-matched controls, but this can be improved by treatment with anti-inflammatory drugs [8]. Despite the beneficial effect of anti-inflammatory drug treatment, however, associations between systemic inflammation and the vasculature is not clearly evident in the literature (which comprises mainly cross-sectional studies) [8,9]. Owing to the fluctuating nature of inflammation in RA, it is possible that assessment of inflammation at a single time point does not accurately reflect the inflammatory insult to the vascular wall over a longer period of time. Characterisation of cumulative inflammation (which incorporates serial measurements of inflammatory markers) might be a better indicator of the patient’s overall inflammatory burden and subsequent risk of developing vascular abnormalities [10].

To the best of our knowledge, investigators in only a few studies have assessed the impact of cumulative inflammation on the vasculature, and they have reported mixed findings. For example, some studies have found an association between cumulative inflammation and the vasculature [11-13], but others have not [14,15]. These contrasting findings are difficult to reconcile, as most of the studies used different methods to determine retrospective or cumulative inflammation. Furthermore, the majority of studies have focused mainly on assessments of vascular morphology, with no study having examined the impact of cumulative inflammation on microvascular endothelial function.

The prevalence of classical CVD risk factors such as hypertension, dyslipidaemia and insulin resistance are increased in RA [16]. We have previously shown that classical CVD risk factors, but not RA-related inflammation, are associated with microvascular and macrovascular endothelial function in RA [9]. Classical CVD risk factors also appear to predict the progression of carotid plaque in patients with RA [17]. It is possible that systemic RA-related inflammation mediates the association between classical CVD risk factors and the vasculature, as some studies have reported that systemic inflammatory markers associate only with vascular abnormalities in the presence of classical CVD risk factors [18].

The objective of the present prospective RA cohort study was to examine the effect of baseline inflammation and classical CVD risk factors on the vasculature following a six-year follow-up period. A secondary objective was to assess associations between cumulative inflammatory burden and the vasculature.

## Methods

### Participants

Four hundred consecutive RA patients were recruited from the rheumatology outpatient clinics of The Dudley Group NHS Foundation Trust in the United Kingdom in 2006. The patients were part of the Dudley Rheumatoid Arthritis Comorbidity Cohort (DRACCO) study, a prospective study examining CVD burden in RA. Detailed characteristics of these patients have been reported previously [19]. Since the initiation of our present study, 78 patients have died due to various causes (cardiac and noncardiac). The remaining 322 were asked to return for a follow-up visit in 2012. From among those patients, 201 agreed to take part in the follow-up study. Only the baseline data (2006) from these patients are reported in this article. All patients met the 1987 revised RA criteria of the American Rheumatism Association [20]. Our study received ethical approval from The Black Country Research Ethics Committee. All participants gave their written informed consent according to the Declaration of Helsinki.

### Protocol for baseline visit

All patients reported to the clinical research facility after a 12-hour overnight fast and underwent a thorough baseline evaluation, including a detailed review of their medical history and hospital records, a physical examination and contemporaneous assessments of height, weight, body mass index, body composition (using a BC-418 Segmental Body Composition Analyzer; Tanita Corporation of America, Arlington Heights, IL, USA), current Disease Activity Score in 28 joints [21] and physical function using the Health Assessment Questionnaire [22]. The Framingham and Reynolds risk scores were calculated as previously described [9]. All medications and their indications were also recorded. Venous blood was collected on the same day as the baseline evaluation, and a wide range of tests were performed. Biochemical and haematological estimations included fasting lipids, complete serum biochemistry, fasting glucose, fasting insulin, C-reactive protein (CRP) and erythrocyte sedimentation rate (ESR). Insulin resistance was assessed by calculating the homeostasis model assessment of insulin resistance and the quantitative insulin sensitivity check index as previously described [23,24]. All biochemical tests were carried out in the Biochemistry Laboratory at Russells Hall Hospital, The Dudley Group NHS Foundation Trust, United Kingdom.

### Protocol for follow-up visit

Patients reported to a temperature-controlled vascular laboratory (22°C) after a 12-hour overnight fast 6 years after the baseline assessment. All patients underwent the same examinations and assessments as in the baseline visit. In addition, patients underwent several functional and morphological vascular assessments, including LDI with iontophoresis of acetylcholine (ACh) and sodium nitroprusside (SNP) (microvascular endothelial function) and assessments of FMD and GTN (macrovascular endothelial function) as well as cIMT (carotid atherosclerosis).

### Microvascular endothelial function

Endothelial function of the microvasculature was assessed noninvasively by a single observer (AS) using LDI (moorLDI2 SIM; Moor Instruments, Axminster, UK) with iontophoresis of 1% ACh (Miochol-E 20 mg; Novartis Pharmaceuticals UK, Horsham, UK) and 1% SNP (Nitroprussiat; Fides Ecopharma, Madrid, Spain) in 2.5 ml of solution containing 0.5% saline. The technique was performed according to established guidelines [25] and has been described in detail previously [5]. Briefly, after a baseline scan, ten scans were recorded during iontophoresis of the vasoactive agents using a 30-μA current, followed by two scans during recovery. This technique has intraobserver coefficients of variation (CVs) for ACh and SNP of 6.5% and 5.9%, respectively, in our laboratory.

### Macrovascular endothelial function

Assessment of macrovascular endothelium-dependent function was performed using FMD with high-resolution ultrasonography of the brachial artery (ACUSON Antares Ultrasound System; Siemens Healthcare Diagnostics, Camberley, UK) according to previously established guidelines [26]. Endothelium-independent responses were examined by administration of a 500-μg sublingual GTN tablet (Alpharma, Barnstaple, UK). The intraobserver CV for the study ultrasonographer (AS) was 10.7% for FMD assessments and 11.8% for GTN assessments. For all vascular tests, endothelial function was expressed as the percentage increase in perfusion or diameter from baseline, and all analyses were carried out offline by AS, who was blinded to the identities of the patients.

### Carotid atherosclerosis

High-resolution ultrasonography of the carotid artery was performed by an experienced ultrasonographer (AS) according to previously established guidelines [27] using a 10-MHz linear array probe attached to the same high-resolution ultrasound scanner used for the FMD assessment. cIMT was defined by determining the thickness between the lines of Pignoli, with the first echogenic line representing the lumen-intima interface and the second line representing the media-adventitia interface [28]. Assessments of cIMT were performed in the far wall, 1 cm proximal to the carotid bulb at sites free of plaque in both the right and left common carotid arteries using the longitudinal scanning plane. Three measurements were taken on each side and averaged to give the mean IMT for the right and left carotid arteries separately. The IMTs from both sides were further averaged to give the overall IMT. The intraobserver CV for AS was 8.6%.

### Cumulative inflammatory burden

Detailed information on all inflammatory markers (CRP and ESR) from the baseline visit to the follow-up visit for each patient was used to calculate cumulative inflammatory burden. A quarterly measurement of CRP and ESR for each year the patient was in the study was used to calculate the areas under the curve (AUCs) for each parameter.

### Statistical analysis

#### Baseline predictors of vascular outcomes

All statistical analyses were performed using IBM SPSS version 20 software (IBM SPSS Inc, Chicago, IL, USA). Initially, six baseline predictors of vascular outcomes were chosen. These included four prevalent classical CVD risk factors in RA (hypertension, dyslipidaemia, insulin resistance and diabetes), along with CRP and ESR. The five vascular outcomes were measurements of ACh, SNP, FMD, GTN and cIMT. In cases where the factor considered was binary and the outcome was continuous, either a *t*-test or Mann–Whitney *U* test was performed, depending on the distribution of the variable, with the results summarised as mean (SE) or median (interquartile range), respectively. In cases where both the variables were continuous, Spearman’s correlation coefficients were calculated. Because so many comparisons were made, the significance of each test was evaluated at both the standard critical value of *P* < 0.05, as well as according to the Bonferroni-corrected version of *P* < 0.001, to minimise the chance of false-positives. The analysis was repeated in a subgroup of patients (*n* = 104) who were free of overt CVD and were not taking nonsteroidal anti-inflammatory drugs (NSAIDs), cyclooxygenase 2 (COX2) inhibitors or anti–tumour necrosis factor α (anti-TNFα) inhibitors.

#### Cumulative inflammatory burden and vascular outcomes

The AUCs for the CRP and ESR data were calculated for the quarterly measurements taken between 2006 and 2012. Because some patients had missing data at the start or the end of the period, the AUCs were dependent on the length of time for which data were available. For this reason, the resulting AUCs were divided by the total period of data collection for each patient to produce average AUCs. The Spearman’s correlation coefficients between these variables and the vascular outcomes were then calculated.

## Results

### Patient characteristics

All 201 patients successfully underwent assessments at both time points. Table 1 displays the patient characteristics. The majority of patients were females with moderate to high disease activity at baseline, but relatively lower disease activity during follow-up. The proportion of patients with hypertension was similar between the baseline and follow-up visits, whereas cases of dyslipidaemia were higher at the follow-up visits. The proportion of patients with insulin resistance decreased slightly at the follow-up visits, but the presence of diabetes increased. There were fewer current smokers at the follow-up visits.

**Table 1:** **Patient characteristics for all rheumatoid arthritis patients at baseline and at follow-up**^**a**^

**Characteristics**	**Baseline (2006)**	**Follow-up (2012)**
General characteristics		
Age (years)	61 (53 to 67)	67 (59 to 73)
Female, *n* (%)	155 (77)	155 (77)
Body mass index (kg/m^2^)	27 (24 to 30)	28 (24 to 32)
Disease characteristics		
Age at RA onset (years)	46 ± 13	–
Disease duration (years)	10 (4 to 18)	16 (11 to 25)
Rheumatoid factor-positive, *n* (%)	148 (74)	148 (74)
Anti-CCP autoantibody-positive, *n* (%)	123 (61)	123 (61)
DAS28 score	4.0 (3.1 to 4.8)	3.1 (2.5 to 4.0)
C-reactive protein (mg/L)	7.5 (4.3 to 16)	3 (2.9 to 8.5)
Erythrocyte sedimentation rate (mm/h)	17 (8 to 30)	12 (5 to 23)
HAQ	1.3 ± 0.9	1.6 ± 0.9
Extraarticular manifestations, *n* (%)^b^	147 (73)	–
Cardiovascular disease risk factors		
Hypertension, *n* (%)	132 (66)	130 (65)
Dyslipidaemia, *n* (%)	115 (57)	158 (79)
Insulin resistance, *n* (%)	65 (32)	53 (26)
Diabetes, *n* (%)	7 (4)	21 (10)
Current smokers	33 (16)	23 (11)
Global cardiovascular disease risk^c^		
Framingham risk score (%)	4.6 ± 5.3	9.2 ± 6.3
Reynolds risk score (%)	6.3 ± 6.4	9.6 ± 8.3
RA medications		
Methotrexate, *n* (%)	128 (64)	122 (61)
Hydroxychloroquine, *n* (%)	36 (18)	50 (25)
Prednisolone, *n* (%)	58 (29)	51 (25)
Prednisolone dose (mg)	6 ± 3	7 ± 8
NSAIDs, *n* (%)	47 (23)	26 (13)
Cyclooxygenase 2 inhibitors, *n* (%)	14 (7)	5 (2.5)
Anti-TNFα therapy, *n* (%)	20 (10)	57 (28)
Tociluzimab, *n* (%)	–	3 (1.5)
Cardiovascular medications		
Antihypertensives, *n* (%)	81 (40)	79 (39)
Antihypercholesterolaemics, *n* (%)	33 (16)	74 (37)
β-blockers, *n* (%)	32 (16)	22 (11)
Calcium channel blockers, *n* (%)	26 (13)	27 (13)

^a^Anti-CCP, anticyclic citrullinated peptide; Anti-TNFα, anti–tumour necrosis factor α; DAS28, Disease Activity Score in 28 joints; HAQ, Health Assessment Questionnaire; NSAID, nonsteroidal anti-inflammatory drug; RA, rheumatoid arthritis. ^b^Data were available for patients at baseline only. Extraarticular manifestations include the presence of nodules, eye abnormalities, systemic vasculitis, erosions, nailfold vasculitis, sicca, pulmonary fibrosis and serositis. ^c^Calculated in patients free of cardiovascular disease and cerebrovascular disease. Results are expressed as median (25th to 75th percentile value), number (%) or mean ± SD.

For medication use, the proportion of patients receiving methotrexate or steroids was similar between the two time points, whereas use of NSAIDs and COX2 inhibitors decreased at follow-up (see Table 1). There was a marked increase in the proportion of patients receiving anti-TNFα medications at follow-up compared to baseline. The proportion of patients receiving antihypertensives, β-blockers and calcium channel blockers remained similar between the two time points. However, use of antihypercholesterolaemics was significantly greater at follow-up.

### Baseline predictors of vascular outcomes in all rheumatoid arthritis patients

The association between baseline factors and vascular outcomes in 2012 is displayed in Additional file 1: Table S1a. The presence of hypertension, dyslipidaemia and insulin resistance all showed some association with reduced ACh (*P* < 0.01). Hypertension and dyslipidaemia showed a similar relationship with SNP (*P* < 0.01). GTN was found to have some association with both hypertension (*P* = 0.002) and insulin resistance (*P* = 0.004), with the presence of either factor resulting in a reduction in GTN. Hypertension and insulin resistance were also found to be associated with increased cIMT (*P* < 0.05). Neither the ESR nor CRP values in 2006 showed any significant associations with any of the 2012 outcomes considered. Diabetes was not shown to be associated significantly with any of the outcomes.

### Baseline predictors of vascular outcomes in rheumatoid arthritis subgroups

As shown in Additional file 1: Table S1b, the results for the patients in the RA subgroups who were free of overt CVD and not taking COX2 inhibitors, NSAIDS or anti-TNFα inhibitors (*n* = 104) are very similar to the analysis of all the RA patients. However, in the subgroups, insulin resistance was no longer associated with ACh and GTN (*P* > 0.2). The *P* values for all significant relationships were not as significant relative to the analysis in all of the patients, but this result was to be expected because the analysis was based on only 50% of the sample size, which gives it less statistical power.

### Effect of smoking pack-years on cardiovascular disease risk factors and vascular outcomes

The smoking pack-years in 2006 were compared to all of the factors and outcomes in the analysis (Table 2). From among all the factors, only insulin resistance was significant, because patients with insulin resistance tended to smoke significantly more than those without (*P* = 0.02). For the outcomes, nonsignificant negative correlations were found for all of the vascular parameters, other than cIMT, which had a significant positive relationship with pack-years (*P* = 0.01).

**Table 2:** **Relationship between smoking pack-years in 2006 and factors and outcomes in main analysis**^**a**^

**Factors in 2006**		**Smoking pack-years (2006)**
Hypertension (2006)	No (*n* = 69)	2 (0 to 18)
	Yes (*n* = 132)	4 (0 to 19)
	*P*-value	0.439
Dyslipidaemia (2006)	No (*n* = 87)	2 (0 to 15)
	Yes (*n* = 114)	3 (0 to 20)
	*P*-value	0.774
Insulin resistance (2006)	No (*n* = 127)	2 (0 to 15)
	Yes (*n* = 65)	10 (0 to 26)
	*P*-value	0.020^b^
Diabetes (2006)	No (*n* = 194)	2 (0 to 18)
	Yes (*n* = 7)	10 (0 to 35)
	*P*-value	0.306
ESR (2006)	Coefficient	0.073
	*P*-value	0.301
CRP (2006)	Coefficient	0.079
	*P*-value	0.268
Ach (2012)	Coefficient	-0.141
	*P*-value	0.050
SNP (2012)	Coefficient	-0.141
	*P*-value	0.050
FMD (2012)	Coefficient	-0.074
	*P*-value	0.313
GTN (2012)	Coefficient	-0.116
	*P*-value	0.136
cIMT (2012)	Coefficient	0.190
	*P*-value	0.014^b^

^a^Ach, acetylcholine; cIMT, carotid intima-media thickness; CRP, C-reactive protein; ESR, erythrocyte sedimentation rate; FMD, flow-mediated dilatation; GTN, glyceryl trinitrate–mediated dilatation; SNP, sodium nitroprusside. ^b^Significant at *P* < 0.05. Data on hypertension, dyslipidaemia, insulin resistance and diabetes are presented as median (25th to 75th quartiles), with *P*-values calculated using the Mann–Whitney *U* test. Data on ESR, CRP, ACh, SNP, FMD, GTN and cIMT are presented as Spearman’s correlation coefficient with associated *P*-values.

### Cumulative inflammatory burden and vascular outcomes

The association of cumulative ESR and CRP with vascular outcomes was examined using Spearman’s correlation. This analysis revealed no evidence of any significant associations between the AUCs of ESR and CRP with the vascular outcomes (see Table 3). The only exception was a weak but statistically significant correlation between the AUC for CRP and the cIMT in 2012 (0.180; *P* = 0.034).

**Table 3:** **Association between cumulative inflammation and the vasculature**^**a**^

**Average AUCs (2006 to 2012)**		**Vascular outcomes in 2012**				
		**ACh**	**SNP**	**FMD**	**GTN**	**cIMT**
ESR	Coefficient	-0.079	-0.036	-0.053	-0.025	0.074
	*P*-value	0.315	0.644	0.515	0.767	0.389
CRP	Coefficient	-0.114	-0.068	-0.027	0.007	0.180
	*P*-value	0.114	0.384	0.732	0.936	0.034^b^

^a^Ach, acetylcholine; AUC, area under the curve; cIMT, carotid intima-media thickness; CRP, C-reactive protein; ESR, erythrocyte sedimentation rate; FMD, flow-mediated dilatation; GTN, glyceryl trinitrate–mediated dilatation; SNP, sodium nitroprusside. ^b^Significant at *P* < 0.05.

## Discussion

The main findings of the present study are that classical CVD risk factors, but not RA disease-related inflammation, predict microvascular endothelium-dependent and endothelium-independent function, macrovascular endothelium-independent function and carotid atherosclerosis in patients with RA who were prospectively followed for six years. These findings were similar in a subgroup of RA patients who were free from overt CVD or were not taking NSAIDs, COX2 inhibitors or anti-TNFα inhibitors, which suggests that the significant effects observed in the present study are generally independent of the effects of heart disease and the three medications that were considered. Furthermore, cumulative inflammation was not associated with microvascular and macrovascular endothelial function, but a weak association was apparent between AUC for CRP and carotid atherosclerosis.

It has previously been hypothesised that elevated inflammation levels contribute to vascular impairments and subsequent CVD risk in RA [3]. However, the present findings after a long follow-up period suggest that inflammatory levels at baseline are not associated with vascular function and morphology. These findings are in agreement with those of a number of other cross-sectional and longitudinal studies of RA [8,9]. Associations between inflammation and the vasculature may be more apparent in patients with high disease activity. Previous studies have shown that patients with a high inflammatory burden at baseline experience greater improvement in vascular function when treated with anti-inflammatory drugs than do patients who have lower baseline inflammation [9,29]. The RA patients in the present cohort had, on average, moderate disease activity, and it is possible that baseline inflammation was not high enough to lead to vascular abnormalities. It is also possible that assessing inflammation at a single time point does not adequately reflect fluctuating inflammation levels, which are characteristic of RA [10]. Therefore, we also calculated the cumulative inflammatory burden and found a weak association of the AUCs for CRP and cIMT.

To the best of our knowledge, there are no studies that have assessed the impact of cumulative inflammation on microvascular and macrovascular endothelial function together with assessments of carotid atherosclerosis in the same group of RA patients. Previous studies have focused mainly on vascular morphology and, similarly to the present study, have reported associations with cumulative inflammation [12,13,30]. In contrast to the present findings, Vaudo and colleagues [11] reported associations between cumulative inflammation and macrovascular endothelium-dependent function. However, macrovascular endothelium-dependent function in their study was considerably lower than it was in the present study (3% vs. 11%, respectively) and reflected a degree of endothelial dysfunction [8]. Given that endothelial dysfunction is an inflammatory process, an association between cumulative inflammation and (depressed) macrovascular endothelium-dependent function could be much more likely in Vaudo and colleagues patient population than in our cohort. Interestingly, studies which have assessed cumulative inflammation for the whole duration of RA reported associations with the vasculature [13,30], whereas calculation of cumulative inflammation for the most recent five years of RA did not [14]. In the current study, cumulative inflammatory burden was calculated only for the six years during which the patients were in the study, which may not reflect the true inflammatory burden. In addition, the increased use of biological therapies, which may have a beneficial effect on CVD, during the period these data were collected might have resulted in lower inflammatory burden and may partly explain our findings. Interestingly however, in our cohort, none of the vascular parameters differed between patients receiving anti-TNFα inhibitor treatment compared to those who did not.

In the microvasculature, the presence of hypertension, dyslipidaemia and insulin resistance at baseline predicted worse microvascular endothelial function. In hypertension, the delicate balance between vasodilators and vasoconstrictors produced by the endothelium is disrupted, with disturbance in the NO pathway leading to predominance of vasoconstrictors such as endothelin 1 (ET-1), which contribute to high blood pressure [31]. Study investigators have reported significant impairments in microvascular endothelium-dependent function in hypertensive patients [32], and we have previously shown that anti-inflammatory treatment may reduce blood pressure in RA patients by improving microvascular endothelium-dependent function [33]. Even though it is still unclear whether endothelial dysfunction is the cause or the consequence of elevated blood pressure, it appears to be an essential factor in hypertension.

In the present cohort, dyslipidaemia was found to predict microvascular endothelial function only. We have previously reported that improvements in microvascular endothelium-dependent function, but not macrovascular endothelium-dependent function, occur alongside a concomitant increase in high-density lipoprotein cholesterol in RA [34]. This suggests that the microvessels may be more susceptible to the adverse effects of dyslipidaemia. Indeed, it has previously been hypothesised that the inflammatory response initiated by hypercholesterolemia occurs in the microvessels before the macrovessels [35]. In particular, increased oxidative stress and elevated inflammatory molecules within the vascular wall lead to a reduction in NO bioavailability along with a concomitant reduction in vasodilatation [5,35]. Intensive treatment with lipid-lowering agents can lower CVD risk in RA [36], possibly as a result of favourable effects in the vasculature.

Insulin resistance was also found to be associated with worse microvascular endothelium-dependent function. Patients with hyperglycaemia have evidence of microvascular endothelial function, mainly due to oxidative stress, as well as increased oxidation of low-density lipoprotein cholesterol [37]. RA has been reported to have a CVD risk burden and vascular profile similar to those of diabetes [38], but diabetes was not shown to associate significantly with any of the vascular outcomes in the present study. However, the mean and median values do seem to imply that RA patients with diabetes have poorer vascular outcomes than those without it. The lack of statistical significance is likely due to low statistical power, owing to the fact that there were few RA patients with diabetes in this cohort (<5% at baseline). Further prospective research examining the effect of diabetes on vascular outcomes in a large sample of RA patients is required.

In the macrovasculature, hypertension and insulin resistance at baseline were both associated with macrovascular endothelium-independent function at follow-up. These findings are similar to the results from our previous cross-sectional study [9]. Assessments of macrovascular endothelium-independent function reflect the integrity of vascular smooth muscle cells [5]. Classical CVD risk factors such as hypertension degrade cyclic guanosine monophosphate (cGMP) [39], a second messenger responsible for the relaxation of vascular smooth muscle cells. In addition, *in vitro* studies have shown that soluble guanylyl cyclase, an enzyme responsible for activating cGMP, has reduced sensitivity to NO in hypertensive rats [40]. This means that even if adequate NO is released from the endothelial cells, abnormalities in smooth muscle cell signalling could still lead to a reduced vasodilatory response.

Similarly to macrovascular endothelium-independent function, RA patients with hypertension and insulin resistance at baseline exhibited greater carotid atherosclerosis at follow-up than patients without these risk factors. Changes in cIMT represent a sequence of events resulting from a decrease in NO bioavailability as well as an increase in ET-1 levels, which over time lead to increased production of proinflammatory cytokines, leucocyte adhesion, activation of thrombotic factors, proliferation of vascular smooth muscle cells and formation of lipid-rich plaques [5]. A previous study of a small number of RA patients (*N* = 47) revealed that patients who experienced a cardiac event during a five-year follow-up period had higher cIMT at baseline than patients who did not have a cardiac event [41]. These findings, together with our current findings, suggest that classical CVD risk factors increase carotid atherosclerosis, which might then result in a higher risk for cardiac events. Indeed, it has recently been shown that cIMT is more sensitive than coronary artery calcification for identifying RA patients at high risk for CVD [42].

In the present study, we also examined the relationship of smoking pack-years with other CVD risk factors and vascular outcomes. RA patients with insulin resistance smoked more than those without insulin resistance. Furthermore, higher smoking pack-years were associated with greater carotid atherosclerosis. Relating this finding back to the original analysis, the significance of the relationship between insulin resistance and carotid atherosclerosis could have been due in part to the effect of smoking. This is because patients with insulin resistance smoke more frequently, and more frequent smokers have higher cIMT. Hence, one would expect the patients with insulin resistance to have higher cIMT due to the effect of smoking.

The strengths of the present study are the inclusion of a large sample of RA survivors from the DRACCO study who were prospectively followed for a long period, as well as the incorporation of several assessments of vascular function and morphology in the microvasculature and the macrovasculature. Such a study design allowed us to investigate specific predictors of vascular outcomes over a protracted time period and provide important additional information on CVD risk. The calculation of cumulative inflammatory burden allowed for the natural fluctuations in disease activity to be taken into account when examining associations with vascular outcomes. However, a limitation of the study is that cumulative inflammation was calculated only for the duration of the study. There is a possibility that inflammation might have been higher early in the patient’s disease course, when optimum treatment had not yet been achieved. Consequently, the calculation of cumulative inflammation in the present study may have underestimated the total inflammatory load of the patient. Unfortunately, the current study assessed only survivors, and several patients experienced cardiovascular events in the observation period but were not included in the analysis. It is possible that different factors might affect vascular function and morphology in such patients. Nevertheless, the findings of the present study suggest that careful screening of CVD risk should include assessment of vascular function and morphology and that such assessments should be used as outcome measures in interventions that aim to reduce CVD risk [43].

We focused on the impact of inflammation and classical CVD risk factors on the vasculature. However, a recently published study in a large RA cohort revealed that several other factors, such as genes (for example, HLA-DRB1 shared epitope alleles), RA-specific factors (for example, autoantibodies and use of systemic steroids) and other factors such as hypothyroidism predicted the presence of CVD in RA [44,45]. Further studies are required to assess the impact of nontraditional CVD risk factors on vascular function and morphology in RA. The findings of the present study specifically highlight the importance of controlling disease activity and classical CVD risk factors in order to halt the progression of vascular abnormalities.

## Conclusion

The present study reveals that classical CVD risk factors may be better long-term predictors of vascular function and morphology than systemic disease-related inflammation in patients with RA. Further studies are needed to confirm whether assessments of vascular function and morphology are predictive of long-term CV outcomes in RA patients.

## Abbreviations

ACh: Acetylcholine; AUC: Area under the curve; BMI: Body mass index; cIMT: Carotid intima-media thickness; CRP: C-reactive protein; CV: Coefficient of variation; CVD: Cardiovascular disease; CVE: Cardiovascular event; DAS28: Disease activity score in 28 joints; ESR: Erythrocyte sedimentation rate; ET-1: Endothelin 1; FMD: Flow-mediated dilatation; GTN: Glyceryl trinitrate–mediated dilatation; HAQ: Health assessment questionnaire; NO: Nitric oxide; NSAID: Nonsteroidal anti-inflammatory drug; RA: Rheumatoid arthritis; SNP: Sodium nitroprusside; TNFα: Tumour necrosis factor α.

## Competing interests

The authors declare that they have no competing interests.

## Authors’ contributions

AS participated in the design of the study, recruited patients, performed the vascular assessments, conducted data analysis and drafted the manuscript. NC helped with data analysis. JH performed the statistical analysis. JPS performed analysis of blood samples. KD participated in the design of the study and recruited patients. GK participated in the design of the study, advised on the analytical approach and helped with drafting the manuscript. All authors read and approved the final manuscript.

## Supplementary Material

Additional file 1: Table S1a Association between baseline factors and vascular outcomes in all rheumatoid arthritis patients. **Table S1b.** Association between baseline factors and vascular outcomes in patients with heart disease or taking cyclooxygenase 2 inhibitors, nonsteroidal anti-inflammatory drugs or anti–tumour necrosis factor α inhibitors excluded. Click here for file

## References

[B1] Maini RN Rheumatoid arthritis: a paradigm of inflammatory disease of the musculoskeletal system Acta Orthop Scand Suppl 1998 15 6 13 9771534

[B2] Kitas GD Erb N Tackling ischaemic heart disease in rheumatoid arthritis Rheumatology (Oxford) 2003 15 607 613 10.1093/rheumatology/keg175 12709534

[B3] Sattar N McCarey DW Capell H McInnes IB Explaining how “high-grade” systemic inflammation accelerates vascular risk in rheumatoid arthritis Circulation 2003 15 2957 2963 10.1161/01.CIR.0000099844.31524.05 14676136

[B4] Stevens RJ Douglas KM Saratzis AN Kitas GD Inflammation and atherosclerosis in rheumatoid arthritis Expert Rev Mol Med 2005 15 1 24 15876361 10.1017/S1462399405009154

[B5] Sandoo A Veldhuijzen van Zanten JJCS Metsios GS Carroll D Kitas GD The endothelium and its role in regulating vascular tone Open Cardiovasc Med J 2010 15 302 312 21339899 10.2174/1874192401004010302 PMC3040999

[B6] Corrado E Rizzo M Coppola G Muratori I Carella M Novo S Endothelial dysfunction and carotid lesions are strong predictors of clinical events in patients with early stages of atherosclerosis: a 24-month follow-up study Coron Artery Dis 2008 15 139 144 10.1097/MCA.0b013e3282f3fbde 18418229

[B7] Lerman A Zeiher AM Endothelial function: cardiac events Circulation 2005 15 363 368 10.1161/01.CIR.0000153339.27064.14 15668353

[B8] Sandoo A Veldhuijzen van Zanten JJCS Metsios GS Carroll D Kitas GD Vascular function and morphology in rheumatoid arthritis: a systematic review Rheumatology 2011 15 2125 2139 10.1093/rheumatology/ker275 21926155

[B9] Sandoo A Kitas GD Carroll D Veldhuijzen van Zanten JJCS The role of inflammation and cardiovascular disease risk on microvascular and macrovascular endothelial function in patients with rheumatoid arthritis: a cross-sectional and longitudinal study Arthritis Res Ther 2012 15 R117 10.1186/ar3847 22594788 PMC3446498

[B10] Sandoo A Kitas G Current perspectives on the assessment of vascular function and morphology in rheumatoid arthritis [Editorial] Int J Clin Rheumatol 2013 15 1 3 10.2217/ijr.12.67

[B11] Vaudo G Marchesi S Gerli R Allegrucci R Giordano A Siepi D Pirro M Shoenfeld Y Schillaci G Mannarino E Endothelial dysfunction in young patients with rheumatoid arthritis and low disease activity Ann Rheum Dis 2004 15 31 35 10.1136/ard.2003.007740 14672888 PMC1754717

[B12] Wållberg-Jonsson S Caidahl K Klintland N Nyberg G Rantapää-Dahlqvist S Increased arterial stiffness and indication of endothelial dysfunction in long-standing rheumatoid arthritis Scand J Rheumatol 2008 15 1 5 10.1080/03009740701633238 18189187

[B13] Crilly MA Kumar V Clark HJ Scott NW Macdonald AG Williams DJ Arterial stiffness and cumulative inflammatory burden in rheumatoid arthritis: a dose–response relationship independent of established cardiovascular risk factors Rheumatology (Oxford) 2009 15 1606 1612 10.1093/rheumatology/kep305 19858120

[B14] Mäki-Petäjä KM Hall FC Booth AD Wallace SML Yasmin Bearcroft PWP Harish S Furlong A McEniery CM Brown J Wilkinson IB Rheumatoid arthritis is associated with increased aortic pulse-wave velocity, which is reduced by anti–tumor necrosis factor-α therapy Circulation 2006 15 1185 1192 10.1161/CIRCULATIONAHA.105.601641 16952987

[B15] Jonsson SW Backman C Johnson O Karp K Lundström E Sundqvist KG Dahlqvist SR Increased prevalence of atherosclerosis in patients with medium term rheumatoid arthritis J Rheumatol 2001 15 2597 2602 11764203

[B16] Kitas GD Gabriel SE Cardiovascular disease in rheumatoid arthritis: state of the art and future perspectives Ann Rheum Dis 2011 15 8 14 10.1136/ard.2010.142133 21109513

[B17] Zampeli E Protogerou A Stamatelopoulos K Fragiadaki K Katsiari CG Kyrkou K Papamichael CM Mavrikakis M Nightingale P Kitas GD Sfikakis PP Predictors of new atherosclerotic carotid plaque development in patients with rheumatoid arthritis: a longitudinal study Arthritis Res Ther 2012 15 R44 10.1186/ar3757 22390577 PMC3446411

[B18] del Rincón I Freeman GL Haas RW O’Leary DH Escalante A Relative contribution of cardiovascular risk factors and rheumatoid arthritis clinical manifestations to atherosclerosis Arthritis Rheum 2005 15 3413 3423 10.1002/art.21397 16255018

[B19] Panoulas VF Douglas KM Milionis HJ Stavropoulos-Kalinglou A Nightingale P Kita MD Tselios AL Metsios GS Elisaf MS Kitas GD Prevalence and associations of hypertension and its control in patients with rheumatoid arthritis Rheumatology (Oxford) 2007 15 1477 1482 10.1093/rheumatology/kem169 17704521

[B20] Arnett FC Edworthy SM Bloch DA McShane DJ Fries JF Cooper NS Healey LA Kaplan SR Liang MH Luthra HS Medsger TA Jr Mitchell DM Neustadt DH Pinals RS Schaller JG Sharp JT Wilder RL Hunder GG The American Rheumatism Association 1987 revised criteria for the classification of rheumatoid arthritis Arthritis Rheum 1988 15 315 324 10.1002/art.1780310302 3358796

[B21] Prevoo ML van ’t Hof MA Kuper HH van Leeuwen MA van de Putte LB van Riel PL Modified disease activity scores that include twenty-eight-joint counts: development and validation in a prospective longitudinal study of patients with rheumatoid arthritis Arthritis Rheum 1995 15 44 48 10.1002/art.1780380107 7818570

[B22] Kirwan JR Reeback JS Stanford Health Assessment Questionnaire modified to assess disability in British patients with rheumatoid arthritis Br J Rheumatol 1986 15 206 209 10.1093/rheumatology/25.2.206 3708236

[B23] Radikova Z Assessment of insulin sensitivity/resistance in epidemiological studies Endocr Regul 2003 15 189 194 14986725

[B24] Katz A Nambi SS Mather K Baron AD Follmann DA Sullivan G Quon MJ Quantitative insulin sensitivity check index: a simple, accurate method for assessing insulin sensitivity in humans J Clin Endocrinol Metab 2000 15 2402 2410 10.1210/jc.85.7.2402 10902785

[B25] Turner J Belch JJ Khan F Current concepts in assessment of microvascular endothelial function using laser Doppler imaging and iontophoresis Trends Cardiovasc Med 2008 15 109 116 10.1016/j.tcm.2008.02.001 18555183

[B26] Corretti MC Anderson TJ Benjamin EJ Celermajer D Charbonneau F Creager MA Deanfield J Drexler H Gerhard-Herman M Herrington D Vallance P Vita J Vogel R International Brachial Artery Reactivity Task Force Guidelines for the ultrasound assessment of endothelial-dependent flow-mediated vasodilation of the brachial artery: a report of the International Brachial Artery Reactivity Task Force J Am Coll Cardiol 2002 15 257 265 A published erratum appears in *J Am Coll Cardiol* 2002, **39:**1082 11788217 10.1016/s0735-1097(01)01746-6

[B27] Touboul PJ Hennerici MG Meairs S Adams H Amarenco P Bornstein N Csiba L Desvarieux M Ebrahim S Hernandez Hernandez R Jaff M Kownator S Naqvi T Prati P Rundek T Sitzer M Schminke U Tardif JC Taylor A Vicaut E Woo KS Mannheim Carotid Intima-Media Thickness and Plaque Consensus (2004–2006–2011): an update on behalf of the Advisory Board of the 3rd, 4th and 5th watching the risk symposia, at the 13th, 15th and 20th European stroke conferences, Mannheim, Germany, 2004, Brussels, Belgium, 2006, and Hamburg, Germany, 2011 Cerebrovasc Dis 2012 15 290 296 10.1159/000343145 23128470 PMC3760791

[B28] Pignoli P Tremoli E Poli A Oreste P Paoletti R Intimal plus medial thickness of the arterial wall: a direct measurement with ultrasound imaging Circulation 1986 15 1399 1406 10.1161/01.CIR.74.6.1399 3536154

[B29] Datta D Ferrell WR Sturrock RD Jadhav ST Sattar N Inflammatory suppression rapidly attenuates microvascular dysfunction in rheumatoid arthritis Atherosclerosis 2007 15 391 395 10.1016/j.atherosclerosis.2006.05.034 16806231

[B30] Park YB Ahn CW Choi HK Lee SH In BH Lee HC Nam CM Lee SK Atherosclerosis in rheumatoid arthritis: morphologic evidence obtained by carotid ultrasound Arthritis Rheum 2002 15 1714 1719 10.1002/art.10359 12124853

[B31] Nadar S Blann AD Lip GY Endothelial dysfunction: methods of assessment and application to hypertension Curr Pharm Des 2004 15 3591 3605 10.2174/1381612043382765 15579056

[B32] Deng LY Li JS Schiffrin EL Endothelium-dependent relaxation of small arteries from essential hypertensive patients: mechanisms and comparison with normotensive subjects and with responses of vessels from spontaneously hypertensive rats Clin Sci (Lond) 1995 15 611 622 7543395 10.1042/cs0880611

[B33] Sandoo A Panoulas VF Toms TE Smith JP Stavropoulos-Kalinoglou A Metsios GS Gasparyan AY Carroll D Veldhuijzen van Zanten JJCS Kitas GD Anti-TNFα therapy may lead to blood pressure reductions through improved endothelium-dependent microvascular function in patients with rheumatoid arthritis J Hum Hypertens 2011 15 699 702 10.1038/jhh.2011.36 21525886

[B34] Sandoo A Veldhuijzen van Zanten JJCS Toms TE Carroll D Kitas GD Anti-TNFα therapy transiently improves high density lipoprotein cholesterol levels and microvascular endothelial function in patients with rheumatoid arthritis: a pilot study BMC Musculoskelet Disord 2012 15 127 10.1186/1471-2474-13-127 22824166 PMC3502606

[B35] Stokes KY Granger DN The microcirculation: a motor for the systemic inflammatory response and large vessel disease induced by hypercholesterolaemia? J Physiol 2005 15 647 653 10.1113/jphysiol.2004.079640 15611017 PMC1665543

[B36] Semb AG Kvien TK DeMicco DA Fayyad R Wun CC LaRosa JC Betteridge J Pedersen TR Holme I Effect of intensive lipid-lowering therapy on cardiovascular outcome in patients with and those without inflammatory joint disease Arthritis Rheum 2012 15 2836 2846 10.1002/art.34524 22576673

[B37] Krentz AJ Clough G Byrne CD Interactions between microvascular and macrovascular disease in diabetes: pathophysiology and therapeutic implications Diabetes Obes Metab 2007 15 781 791 10.1111/j.1463-1326.2007.00670.x 17924862

[B38] Peters MJ van Halm VP Voskuyl AE Smulders YM Boers M Lems WF Visser M Stehouwer CD Dekker JM Nijpels G Heine R Dijkmans BA Nurmohamed MT Does rheumatoid arthritis equal diabetes mellitus as an independent risk factor for cardiovascular disease? A prospective study Arthritis Rheum 2009 15 1571 1579 10.1002/art.24836 19877093

[B39] Yugar-Toledo JC Ferreira-Melo SE Consolim-Colombo FM Irigoyen MC Coelho OR Moreno H Jr Cyclic guanosine monophosphate phosphodiesterase-5 inhibitor promotes an endothelium NO-dependent-like vasodilation in patients with refractory hypertension Nitric Oxide 2007 15 315 321 10.1016/j.niox.2006.12.004 17276107

[B40] Kojda G Kottenberg K Hacker A Noack E Alterations of the vascular and the myocardial guanylate cyclase/cGMP-system induced by long-term hypertension in rats Pharm Acta Helv 1998 15 27 35 10.1016/S0031-6865(97)00044-7 9708036

[B41] Gonzalez-Juanatey C Llorca J Martin J Gonzalez-Gay MA Carotid intima-media thickness predicts the development of cardiovascular events in patients with rheumatoid arthritis Semin Arthritis Rheum 2009 15 366 371 10.1016/j.semarthrit.2008.01.012 18336869

[B42] Corrales A Parra JA González-Juanatey C Rueda-Gotor J Blanco R Llorca J González-Gay MA Cardiovascular risk stratification in rheumatic diseases: carotid ultrasound is more sensitive than Coronary Artery Calcification Score to detect subclinical atherosclerosis in patients with rheumatoid arthritis Ann Rheum Dis 2013 15 1764 1770 10.1136/annrheumdis-2013-203688 23852762

[B43] Metsios GS Stavropoulos-Kalinoglou A Veldhuijzen van Zanten JJCS Nightingale P Sandoo A Dimitroulas T Kitas GD Koutedakis Y Individualised exercise improves endothelial function in patients with rheumatoid arthritis Ann Rheum Disin press. doi:10.1136/annrheumdis-2013-203291 10.1136/annrheumdis-2013-203291 23904472

[B44] Amaya-Amaya J Sarmiento-Monroy JC Mantilla RD Pineda-Tamayo R Rojas-Villarraga A Anaya JM Novel risk factors for cardiovascular disease in rheumatoid arthritis Immunol Res 2013 15 267 286 10.1007/s12026-013-8398-7 23584985

[B45] Palomino-Morales R Gonzalez-Juanatey C Vazquez-Rodriguez TR Miranda-Filloy JA Llorca J Martin J Gonzalez-Gay MA Interleukin-6 gene -174 promoter polymorphism is associated with endothelial dysfunction but not with disease susceptibility in patients with rheumatoid arthritis Clin Exp Rheumatol 2009 15 964 970 20149313

