# Supplementary material for: Classical cardiovascular disease risk factors associate with vascular function and morphology in rheumatoid arthritis: a six-year prospective study
Source: Arthritis Res Ther. 2013 Dec 2;15(6):R203. doi: 10.1186/ar4396 (PMC3979105; doi:10.1186/ar4396)
Supplement: Additional file 1: Table S1a — Association between baseline factors and vascular outcomes in all rheumatoid arthritis patients. Table S1b. Association between baseline factors and vascular outcomes in patients with heart disease or taking cyclooxygenase 2 inhibitors, nonsteroidal anti-inflammatory drugs or anti–tumour necrosis factor α inhibitors excluded. [file ar4396-S1.doc]

Table S1a. Association between baseline factors and vascular outcomes in all RA patients

| **Factors in 2006** | | **Vascular Outcomes in 2012** | | | | |
| --- | --- | --- | --- | --- | --- | --- |
|  |  | **ACh** | **SNP** | **FMD** | **GTN** | **cIMT** |
| Hypertension | No *(N=69)* | 305 (132-488) | 161 (90-267) | 11.6 (0.9) | 24.5 (1.2) | 0.66 (0.02) |
|  | Yes *(N=132)* | 184 (78-343) | 116 (44-198) | 10.0 (0.6) | 20.1 (0.8) | 0.71 (0.01) |
|  | *p-Value* | ***<0.001***** | ***0.004**** | *0.159* | ***0.002**** | ***0.049**** |
| Dyslipidemia | No *(N=86)* | 238 (149-467) | 159 (81-250) | 10.6 (0.8) | 22.0 (1.0) | 0.70 (0.02) |
|  | Yes *(N=115)* | 188 (76-344) | 115 (44-190) | 10.6 (0.7) | 21.5 (1.0) | 0.69 (0.01) |
|  | *p-Value* | ***0.007**** | ***0.007**** | *0.982* | *0.722* | *0.747* |
| Insulin Resistance | No *(N=127)* | 241 (136-444) | 143 (72-240) | 11.3 (0.7) | 23.3 (0.9) | 0.67 (0.01) |
|  | Yes *(N=65)* | 153 (71-320) | 116 (48-211) | 9.4 (0.9) | 19.0 (1.1) | 0.73 (0.02) |
|  | *p-Value* | ***0.003**** | *0.137* | *0.103* | ***0.004**** | ***0.021**** |
| Diabetes | No *(N=194)* | 202 (102-392) | 132 (63-222) | 10.8 (0.5) | 21.9 (0.7) | 0.69 (0.01) |
|  | Yes *(N=7)* | 145 (125-327) | 55 (38-160) | 5.5 (1.2) | 16.2 (3.3) | 0.81 (0.06) |
|  | *p-Value* | *0.557* | *0.103* | *0.052* | *0.163* | *0.105* |
| ESR | Coefficient | -0.035 | -0.039 | -0.026 | -0.061 | 0.107 |
|  | *p-Value* | *0.633* | *0.585* | *0.721* | *0.436* | *0.171* |
| CRP | Coefficient | 0.009 | -0.056 | -0.050 | -0.155 | 0.069 |
|  | *p-Value* | *0.902* | *0.436* | *0.495* | *0.047** | *0.380* |

Data displayed as:

“Median (Quartiles)”, with p-values from Mann-Whitney test

“Mean (SE)”, with p-values from t-test

“Spearman’s Correlation Coefficient”, with associated p-values

*Significant at p<0.05, **Significant after Bonferroni correction for 30 comparisons (p<0.001)

ACh = acetylcholine; CRP = C-reactive protein; ESR = erythrocyte sedimentation rate; FMD = flow mediated dilatation; GTN = glyceryl trinitrate mediated dilatation; cIMT = carotid intima media thickness; SNP = sodium nitroprusside

Table S1b. Association between baseline factors and vascular outcomes in patients with Heart Disease or taking COXIB, NSAID or Anti-TNF excluded

| **Factors in 2006** | | **Vascular Outcomes in 2012** | | | | |
| --- | --- | --- | --- | --- | --- | --- |
|  |  | **ACh** | **SNP** | **FMD** | **GTN** | **cIMT** |
| Hypertension | No *(N=39)* | 234 (109-586) | 171 (79-311) | 13.5 (1.2) | 23.8 (1.5) | 0.65 (0.03) |
|  | Yes *(N=59)* | 176 (78-285) | 118 (36-191) | 10.5 (1.1) | 18.4 (1.1) | 0.73 (0.02) |
|  | *p-Value* | ***0.046**** | ***0.032**** | *0.064* | ***0.004**** | ***0.011**** |
| Dyslipidemia | No *(N=48)* | 226 (135-420) | 165 (75-277) | 11.1 (1.0) | 21.1 (1.3) | 0.70 (0.02) |
|  | Yes *(N=50)* | 142 (70-293) | 118 (33-176) | 12.4 (1.2) | 20.5 (1.4) | 0.70 (0.02) |
|  | *p-Value* | ***0.020**** | ***0.033**** | *0.428* | *0.770* | *0.862* |
| Insulin Resistance | No *(N=64)* | 204 (113-414) | 132 (58-222) | 12.3 (1.0) | 21.5 (1.1) | 0.67 (0.02) |
|  | Yes *(N=29)* | 176 (82-308) | 149 (71-234) | 10.7 (1.4) | 18.5 (1.7) | 0.76 (0.03) |
|  | *p-Value* | *0.278* | *0.864* | *0.364* | *0.160* | ***0.014**** |
| Diabetes | No *(N=95)* | 197 (91-372) | 132 (56-223) | 11.9 (0.8) | 21.0 (1.0) | 0.69 (0.02) |
|  | Yes *(N=3)* | 128 (NA) | 75 (NA) | 6.6 (2.2) | 12.7 (3.3) | 0.82 (0.09) |
|  | *p-Value* | *0.725* | *0.287* | *0.254* | *0.189* | *0.157* |
| ESR | Coefficient | -0.076 | -0.173 | 0.086 | -0.089 | 0.089 |
|  | *p-Value* | *0.460* | *0.089* | *0.407* | *0.416* | *0.421* |
| CRP | Coefficient | -0.030 | -0.151 | 0.067 | -0.077 | -0.004 |
|  | *p-Value* | *0.772* | *0.139* | *0.515* | *0.480* | *0.974* |

Data displayed as:

“Median (Quartiles)”, with p-values from Mann-Whitney test

“Mean (SE)”, with p-values from t-test

“Spearman’s Correlation Coefficient”, with associated p-values

*Significant at p<0.05, **Significant after Bonferroni correction for 30 comparisons (p<0.001)

NA = Quartiles not reported due to small sample size

ACh = acetylcholine; Anti-TNF = anti-tumour necrosis factor alpha; CRP = C-reactive protein; COXIB = cyclooxygenase II inhibitors; ESR = erythrocyte; NSAID = non steroidal anti-inflammatory dug; sedimentation rate; FMD = flow mediated dilatation; GTN = glyceryl trinitrate mediated dilatation; cIMT = carotid intima media thickness; SNP = sodium nitroprusside.
